# Supplementary material for: Evolution of sexual dimorphism and Rensch’s rule in the beetle genus Limnebius (Hydraenidae): is sexual selection opportunistic?
Source: PeerJ. 2017 Mar 7;5:e3060. doi: 10.7717/peerj.3060 (PMC5344018; doi:10.7717/peerj.3060)
Supplement: Supplemental Information 1 [file peerj-05-3060-s001.pdf]

Evolution of sexual dimorphism and Rensch's rule in the beetle genus *Limnebius* (Hydraenidae) - is sexual selection opportunistic?

Supporting information

Table S1. Studied species of *Limnebius*, with species groups, authors, and measures per species. lm, lf and lg, length of males, females and male genitalia respectively; Nm, Nf and Ng, number of measured males, females and male genitalia; stv.lm, stv.lf and stv.lg, standard deviation of measured males, females and male genitalia (for less than seven specimens the total range is given); per, perimeter of the male genitalia; fd, fractal dimension of the male genitalia.

Table S2. Specimens used in the molecular phylogeny, with country, voucher, species group according to Rudoy et al. (2016) and GenBank accession numbers (from Rudoy & Ribera, 2016).

Table S3. Reconstructed values of the measured variables in all branches of the phylogeny and estimated measurements of SSD. See Fig. S4 for the location of the branch numbers in the phylogeny. The raw values of male and female body size of the initial and final nodes of each branch are given, together with the amount of phenotypic change and the change measured in darwins (x10^6). In pink, composite branch after deleting terminals with missing males. In yellow, estimated values of *lf*.

Evolution of the sexual dimorphism in the beetle genus Limnebius (Hydraenidae) - is sexual selection opportunistic?

Andrey Rudoy & Ignacio Ribera

Table S1

Studied species of *Limnebius*, with species groups, authors, voucher of the speciens used in then molecular phylogeny, and measures per species.
lm, lf and lg, length of males, females and male genitalia respectively; Nm, Nf and Ng, number of measured males, females and male genitalia; stv.lm, stv.lf and stv.lg, standard deviation of measured males, females and male genitalia; per, perimeter of the male genitalia; fd, fractal dimension of the male genitalia; SSCt, secondary sexual characters of the metatibiae (0 none, 1 with setae, 2 widened, 3 with distal part enlarged); SSCa, secondary sexual characters of the VI abdominal ventrite (0 none, 1 with setae, 2 with a protuberance).

No	genus	subgenus	species group	species	author and year	voucher (molecular phylog	lm	lf	lg	Nm	Nf	Ng	stv.lm	stv.lf	stv.lg	per	fd	SSCt	SSCa
1	*Laeliaena*			*sahlbergi*	Champion, 1920	MNCN-HI19	1.21	?	0.34	3	0	3	1.20-1.22	-	0.32-0.35	3.003	1.284	0	0
2	*Laeliaena*			*sichuanensis*	Jäch, 1995		1.42	1.43	0.39	7	46	7	0.065	0.055	0.0140	2.836	1.256	0	0
3	*Laeliaena*			*sparsa*	Sahlberg, 1900		1.11	?	0.38	2	0	2	1.10-1.11	-	0.36-0.39	2.344	1.232	0	0
4	*Limnebius*	*Bilimneus*		*acupunctus*	Perkins, 2004	MNCN-AI582	[0.85]	?	[0.21]	0	0	0	-	-	-	-	-	0	0
5	*Limnebius*	*Bilimneus*		*angustipennis*	d'Orchymont, 1932		0.84	?	0.29	1	0	1	-	-	-	2.262	1.176	0	0
6	*Limnebius*	*Bilimneus*		*arabicus*	Balfour-Browne, 1951		0.76	0.85	0.23	8	8	1	0.024	0.023	-	2.316	1.174	0	0
7	*Limnebius*	*Bilimneus*		*atomus*	(Duftschmid, 1805)	MNCN-AI1217	0.99	1.06	0.41	12	6	9	0.031	0.99-1.12	0.0154	2.340	1.176	0	0
8	*Limnebius*	*Bilimneus*		*boukali*	Jäch, 1993		0.94	1.06	0.25	14	5	4	0.041	1.03-1.1	0.24-0.25	2.438	1.186	0	0
9	*Limnebius*	*Bilimneus*		*clavatus*	Pu, 1951		0.81	?	0.21	2	0	2	0.80-0.81	-	0.20-0.21	2.457	1.164	0	0
10	*Limnebius*	*Bilimneus*		*cruzei*	Jäch, 1982		0.87	1.03	0.32	1	2	1	-	1-1.05	-	2.106	1.181	0	0
11	*Limnebius*	*Bilimneus*		*curidius*	d'Orchymont, 1941		0.98	1.07	0.33	9	7	2	0.030	0.035	0.32-0.34	2.392	1.172	0	0
12	*Limnebius*	*Bilimneus*		*dioscoridus*	Jäch & Delgado, 2012	IBE-RA728	0.84	0.98	0.23	4	4	1	0.74-0.77	0.83-0.86	0.22-0.24	2.310	1.175	0	0
13	*Limnebius*	*Bilimneus*		*endroedyi*	Perkins, 2015	IBE-AN78	0.86	1.08	0.41	1	1	1	-	-	-	2.177	1.173	0	0
14	*Limnebius*	*Bilimneus*		*evanescens*	Kiesenwetter, 1866	MNCN-AI915	0.88	1.01	0.32	36	14	32	0.040	0.039	0.0144	2.250	1.166	0	0
15	*Limnebius*	*Bilimneus*		*extraneus*	d'Orchymont, 1938	IBE-RA89	0.84	0.95	0.37	17	10	15	0.044	0.053	0.0150	2.347	1.174	0	0
16	*Limnebius*	*Bilimneus*		*feuerborni*	d'Orchymont, 1932	IBE-AR11	0.83	?	0.25	1	0	1	-	-	-	2.477	1.165	0	0
17	*Limnebius*	*Bilimneus*		*kelaniyae*	Jäch, 1982		0.82	?	0.36	1	0	1	-	-	-	2.456	1.184	0	0
18	*Limnebius*	*Bilimneus*		*kwangtungensis*	Pu, 1936		0.88	?	0.29	3	0	3	0.84-0.90	-	0.28-0.30	2.309	1.170	0	0
19	*Limnebius*	*Bilimneus*		*kweichowensis*	Pu, 1951		0.96	1.04	0.47	7	14	2	0.019	0.042	0.46-0.47	2.610	1.181	0	0
20	*Limnebius*	*Bilimneus*		*loeblorum*	Jäch, 1993		0.94	?	0.29	1	0	1	-	-	-	2.394	1.169	0	0
21	*Limnebius*	*Bilimneus*		*myrmidon*	Rey, 1883	MNCN-AI703	0.89	0.97	0.23	34	22	14	0.041	0.026	0.0072	2.522	1.165	0	0
22	*Limnebius*	*Bilimneus*		*nakanei nakanei*	Jäch & Matsui, 1994	MNCN-AC30	0.86	0.98	0.23	6	10	4	0.84-0.87	0.88-0.98	0.23-0.30	2.359	1.171	0	0
23	*Limnebius*	*Bilimneus*		*nanus*	Jäch, 1993		0.84	0.92	0.25	1	0	1	-	-	-	2.268	1.164	0	0
24	*Limnebius*	*Bilimneus*		*oblongus*	Rey, 1883	MNCN-AI942	1.01	1.08	0.43	13	1	11	0.042	0.023	0.0097	2.343	1.176	0	0
25	*Limnebius*	*Bilimneus*		*pararabicus*	Jäch & Delgado, 2013	IBE-RA761	0.82	1.00	0.26	9	1	8	0.035	-	0.0116	2.217	1.177	0	0
26	*Limnebius*	*Bilimneus*		*perparvulus*	Rey, 1884	MNCN-AI428	0.91	1.00	0.44	47	37	21	0.042	0.048	0.0230	2.246	1.180	0	0
27	*Limnebius*	*Bilimneus*		*pollex*	Jäch & Delgado, 2013	IBE-RA1019	0.93	?	0.44	1	0	1	-	-	-	2.263	1.159	0	0
28	*Limnebius*	*Bilimneus*		*rufipennis*	Régimbart, 1903		0.80	0.91	0.25	19	9	9	0.045	0.022	0.0137	2.316	1.167	0	0
29	*Limnebius*	*Bilimneus*		sp India1		IBE-AF193	0.83	1.38	0.31	2.00	4	3	1.26-1.31	1.34-1.4	0.49-0.51	2.200	1.168	0	0
30	*Limnebius*	*Bilimneus*		sp India2			0.99	?	0.39	1	0	1	-	-	-	-	-	0	0
31	*Limnebius*	*Bilimneus*		sp Bhutan		MNCN-AI1271	0.83	1.06	0.31	1	4	1	-	0.99-1.16	-	2.200	1.170	0	0
32	*Limnebius*	*Bilimneus*		*taiwanensis*	Jäch, 1993		0.83	1.00	0.22	18	12	9	0.048	0.042	0.0121	2.246	1.166	0	0
33	*Limnebius*	*Bilimneus*		*thienemanni*	d'Orchymont, 1932		0.98	1.15	0.24	4	6	3	0.93-1.04	1.06-1.18	0.23-0.26	2.148	1.175	0	0
34	*Limnebius*	*Bilimneus*		*wewalkai*	Jäch & Delgado, 2010	IBE-RA108	0.83	0.91	0.36	13	10	7	0.032	0.043	0.0086	2.277	1.184	0	0
35	*Limnebius*	*Limnebius*	gracilipes	*canariensis*	d'Orchymont, 1938		1.66	1.37	0.49	3	1	3	1.56-1.75	-	0.47-0.50	5.324	1.335	0	2
36	*Limnebius*	*Limnebius*	gracilipes	*cordobanus*	d'Orchymont, 1938	MNCN-AI1024/IBE-PA275	0.92	1.01	0.33	24	14	11	0.057	0.049	0.0100	3.850	1.252	0	0
37	*Limnebius*	*Limnebius*	gracilipes	*fallaciosus*	Ganglbauer, 1904	IBE-RA1121	1.31	1.40	0.42	22	28	4	0.035	0.041	0.41-0.45	7.254	1.335	0	2
38	*Limnebius*	*Limnebius*	gracilipes	*gracilipes*	Wollaston, 1864	MNCN-AI1094	1.32	1.50	0.40	15	0	5	0.054	0.032	0.37-0.42	5.130	1.323	0	2
39	*Limnebius*	*Limnebius*	gracilipes	*paganettii*	Ganglbauer, 1904		1.77	1.41	0.50	27	9	10	0.073	0.058	0.0145	7.446	1.374	0	2
40	*Limnebius*	*Limnebius*	nitidus-mundus	*attalensis*	Jäch, 1993		1.04	?	0.28	2	0	2	1.04-1.09	-	0.28-0.32	2.878	1.201	0	0
41	*Limnebius*	*Limnebius*	nitidus-mundus	*distinguendus*	Ferro, 1989		1.15	?	0.30	2	0	2	1.10-1.20	-	0.29-0.32	2.436	1.161	0	0
42	*Limnebius*	*Limnebius*	nitidus-mundus	*ferroi*	Jäch, 1993		1.00	1.11	0.33	18	8	6	0.034	0.013	0.32-0.34	2.266	1.194	0	0
43	*Limnebius*	*Limnebius*	nitidus-mundus	*fontinalis*	Balfour-Browne, 1951		1.14	1.35	0.44	2	2	2	1.13-1.15	1.31-1.39	0.43-0.45	2.349	1.183	0	0
44	*Limnebius*	*Limnebius*	nitidus-mundus	*mundus*	Baudi di Selve, 1864		1.02	?	0.24	1	0	1	-	-	-	2.456	1.198	0	0
45	*Limnebius*	*Limnebius*	nitidus-mundus	*murcus*	d'Orchymont, 1945		0.95	1.06	0.33	1	1	1	-	-	-	2.380	1.190	0	0
46	*Limnebius*	*Limnebius*	nitidus-mundus	*murentius*	d'Orchymont, 1945	IBE-AN53	1.09	1.15	0.32	10	4	10	0.041	1.1-1.18	0.0106	2.379	1.187	0	0

47	*Limnebius*	*Limnebius*	nitidus-mundus	*nigritus*	Balfour-Browne, 1956		1.09	1.15	0.30	1	0	1	-	-	-	2.343	1.177	0	0
48	*Limnebius*	*Limnebius*	nitidus-mundus	*paranuristanus*	Ferro, 1989		1.08	0.95	0.29	4	1	4	1.07-1.10	-	0.28-0.31	2.404	1.189	0	0
49	*Limnebius*	*Limnebius*	nitidus-mundus	*sanctimontis*	Jäch, 1993		1.12	1.26	0.39	8	15	3	0.030	0.044	0.38-0.41	2.236	1.195	0	0
50	*Limnebius*	*Limnebius*	nitidus-mundus	sp Arabia			1.31	1.38	0.50	2	0	2	0.99-1.05	-	0.49-0.51	2.704	1.178	0	0
51	*Limnebius*	*Limnebius*	nitidus-nitiduloides	*alibeii*	Hernando, Aguilera & Ribera, 1999	IBE-RA90	2.16	1.99	1.09	4	1	4	2.11-2.20	-	1.07-1.11	7.503	1.365	1	2
52	*Limnebius*	*Limnebius*	nitidus-nitiduloides	*calabricus*	Jäch, 1993		1.87	1.84	0.66	6	8	3	1.83-1.93	0.046	0.59-0.70	6.904	1.360	1	2
53	*Limnebius*	*Limnebius*	nitidus-nitiduloides	*crassipes*	Kuwert, 1890	IBE-RA68	2.33	1.92	0.73	13	11	5	0.086	0.086	0.68-0.77	8.379	1.410	1	2
54	*Limnebius*	*Limnebius*	nitidus-nitiduloides	*fretalis*	Peyerimhoff, 1913	MNCN-AI1307	2.39	2.12	1.21	31	56	23	0.058	0.076	0.0446	8.116	1.377	1	2
55	*Limnebius*	*Limnebius*	nitidus-nitiduloides	*hispanicus*	d'Orchymont, 1941	MNCN-AI1309	2.06	1.90	0.71	3	2	3	2.02-2.04	1.8-2	0.69-0.73	8.625	1.368	1	2
56	*Limnebius*	*Limnebius*	nitidus-nitiduloides	*levantinus*	Jäch, 1993	IBE-RA731	2.00	1.82	0.66	10	13	7	0.049	0.064	0.0300	9.309	1.424	1	2
57	*Limnebius*	*Limnebius*	nitidus-nitiduloides	*nitiduloides*	Baudi di Selve, 1872	IBE-AR16	2.45	2.22	1.00	9	4	7	0.121	2.19-2.23	0.0468	8.386	1.403	1	2
58	*Limnebius*	*Limnebius*	nitidus-nitiduloides	*schoenmanni*	Jäch, 1993	IBE-RA1000	2.23	?	0.59	5	0	5	2.13-2.40	-	0.68-0.73	7.876	1.388	1	2
59	*Limnebius*	*Limnebius*	nitidus-nitiduloides	*simplex*	Baudi di Selve, 1882	MNCN-AC39	1.96	1.96	0.68	4	2	4	1.84-2.08	0.075	0.66-0.70	8.125	1.387	1	2
60	*Limnebius*	*Limnebius*	nitidus-nitiduloides	*simulans*	d'Orchymont, 1940		2.00	1.72	0.73	1	2	1	-	1.69-1.74	-	7.496	1.397	1	2
61	*Limnebius*	*Limnebius*	nitidus-nitiduloides	*spinosus*	Jäch, 1993	IBE-AN54	1.98	1.83	0.65	10	17	3	0.071	0.065	0.60-0.68	7.774	1.394	1	2
62	*Limnebius*	*Limnebius*	nitidus-nitiduloides	*theryi*	Guillebeau, 1891	MNCN-AI950	2.24	1.92	0.84	5	4	4	2.22-2.39	1.94-1.97	0.83-0.85	10.074	1.371	1	2
63	*Limnebius*	*Limnebius*	nitidus-nitidus	*bacchus*	Balfour-Browne, 1979	IBE-PA274	1.26	1.27	0.33	5	4	5	1.11-1.32	1.03-1.1	0.31-0.36	4.546	1.279	0	0
64	*Limnebius*	*Limnebius*	nitidus-nitidus	*corfidius*	d'Orchymont, 1945	IBE-AN33	1.14	1.19	0.31	10	0	10	0.021	0.078	0.0090	4.242	1.250	0	0
65	*Limnebius*	*Limnebius*	nitidus-nitidus	*corybus*	d'Orchymont, 1945	MNCN-AI1280	1.22	1.25	0.34	20	15	20	0.053	0.059	0.3380	4.298	1.246	0	0
66	*Limnebius*	*Limnebius*	nitidus-nitidus	*graecus*	Jäch, 1993		1.31	?	0.48	7	0	7	0.046	-	0.0080	2.732	1.186	0	0
67	*Limnebius*	*Limnebius*	nitidus-nitidus	*grandicollis*	Wollaston, 1854		1.47	1.47	0.50	2	9	2	1.47-1.44	0.076	0.44-0.50	3.476	1.185	0	0
68	*Limnebius*	*Limnebius*	nitidus-nitidus	*hieronymi*	Vorst, 2006	IBE-AR22	1.28	1.28	0.38	25	10	24	0.054	0.045	0.0100	2.668	1.178	0	0
69	*Limnebius*	*Limnebius*	nitidus-nitidus	*ibericus*	Balfour-Browne, 1979	IBE-RA88	1.21	1.26	0.43	6	11	5	1.21-1.30	0.072	0.40-0.45	4.402	1.261	0	0
70	*Limnebius*	*Limnebius*	nitidus-nitidus	*kocheri*	Balfour-Browne, 1979	IBE-RA91	1.33	1.45	0.32	12	15	12	0.048	0.077	0.0167	3.554	1.243	0	2
71	*Limnebius*	*Limnebius*	nitidus-nitidus	*minoricensis*	Jäch, Valladares & García-Avilés, 1996	IBE-AR23	1.24	1.40	0.42	27	44	27	0.038	0.062	0.0250	2.624	1.186	0	0
72	*Limnebius*	*Limnebius*	nitidus-nitidus	*nitigeus*	d'Orchymont, 1945		1.38	?	0.51	4	0	4	1.33-1.46	-	0.50-0.52	3.110	1.201	0	0
73	*Limnebius*	*Limnebius*	nitidus-nitidus-cplx	*aguilerai*	Ribera & Millán, 1998	MNCN-AI910	1.38	1.37	0.52	5	5	5	1.30-1.53	1.29-1.43	0.49-0.54	4.604	1.298	0	0
74	*Limnebius*	*Limnebius*	nitidus-nitidus-cplx	*gerhardti*	L. Heyden, 1870	MNCN-AI891	1.42	1.38	0.46	20	1	20	0.073	-	0.0146	5.102	1.295	0	0
75	*Limnebius*	*Limnebius*	nitidus-nitidus-cplx	*hilaris*	Balfour-Browne, 1979	MNCN-AI919	1.35	1.34	0.48	24	20	24	0.043	0.059	0.0200	4.642	1.279	0	0
76	*Limnebius*	*Limnebius*	nitidus-nitidus-cplx	*irmelae*	Jäch, 1993	MNCN-AI949	1.43	1.41	0.57	2	3	2	1.37-1.47	1.38-1.45	0.57-0.58	4.410	1.271	0	0
77	*Limnebius*	*Limnebius*	nitidus-nitidus-cplx	*maurus*	Balfour-Browne, 1979	MNCN-AI1317	1.44	1.41	0.49	86	52	84	0.066	0.054	0.0186	4.302	1.284	0	0
78	*Limnebius*	*Limnebius*	nitidus-nitidus-cplx	*millani*	Ribera & Hernando, 1998	MNCN-AI920	1.36	1.39	0.48	4	2	2	1.32-1.38	1.36-1.41	0.47-0.49	5.448	1.307	0	0
79	*Limnebius*	*Limnebius*	nitidus-nitidus-cplx	*monfortei*	Fresneda & Ribera, 1998	IBE-AF199	1.44	1.45	0.45	3	2	3	1.43-1.44	1.41-1.47	0.45-0.47	4.888	1.298	0	0
80	*Limnebius*	*Limnebius*	nitidus-nitidus-cplx	*montanus*	Balfour-Browne, 1979	MNCN-AI936	1.36	1.36	0.64	5	3	5	1.13-1.44	1.33-1.37	0.59-0.67	5.264	1.300	0	0
81	*Limnebius*	*Limnebius*	nitidus-nitidus-cplx	*nitidus*	(Marsham, 1802)	MNCN-AI377	1.40	1.38	0.51	24	14	24	0.050	0.032	0.0139	5.060	1.297	0	0
82	*Limnebius*	*Limnebius*	nitidus-nitidus-cplx	*nitifarus*	d'Orchymont, 1938		1.48	?	0.49	1	0	1	-	-	-	5.280	1.307	0	0
83	*Limnebius*	*Limnebius*	nitidus-nitidus-cplx	*ordunyai*	Fresneda & Ribera, 1998	MNCN-AI848	1.30	1.30	0.45	10	6	8	0.062	1.24-1.37	0.0100	4.686	1.282	0	0
84	*Limnebius*	*Limnebius*	nitidus-punctatus	*ignarus*	Balfour-Browne, 1979	MNCN-AI993	1.11	1.10	0.39	4	4	3	0.97-1.17	1.07-1.16	0.38-0.41	5.638	1.339	0	2
85	*Limnebius*	*Limnebius*	nitidus-punctatus	*kamali*	Sáinz-Cantero & Bennas, 2006		1.30	1.46	0.56	1	3	1	-	1.42-1.48	-	6.823	1.367	0	2
86	*Limnebius*	*Limnebius*	nitidus-punctatus	*mucronatus*	Baudi di Selve, 1872	MNCN-AI704	1.18	1.29	0.37	40	33	16	0.049	0.061	0.0095	6.050	1.303	0	2
87	*Limnebius*	*Limnebius*	nitidus-punctatus	*pilicauda*	Guillebeau, 1896	IBE-AR60	1.51	1.27	0.46	44	29	35	0.079	0.073	0.0187	7.778	1.362	0	2
88	*Limnebius*	*Limnebius*	nitidus-punctatus	*punctatus*	Wollaston, 1864		1.46	1.55	0.44	1	1	1	-	-	-	6.236	1.303	0	2
89	*Limnebius*	*Limnebius*	nitidus-punctatus	*similis*	Wollaston, 1865		1.25	1.40	0.35	3	2	2	1.20-1.27	1.37-1.43	0.34-0.35	6.110	1.326	0	2
90	*Limnebius*	*Limnebius*	nitidus-punctatus	*zaerensis*	Hernando, Aguilera & Ribera, 2008	MNCN-AC14	1.31	?	0.51	1	0	1	-	-	-	7.201	1.349	0	2
91	*Limnebius*	*Limnebius*	nitidus-truncatellus	*mesatlanticus*	Théry, 1933	MNCN-AI913	2.04	1.91	0.81	19	15	19	0.072	0.089	0.0140	8.116	1.387	3	2
92	*Limnebius*	*Limnebius*	nitidus-truncatellus	*truncatellus*	(Thunberg, 1794)	MNCN-AI451	2.38	1.98	0.70	126	160	104	0.095	0.072	0.0300	9.424	1.395	3	2
93	*Limnebius*	*Limnebius*	parvulus-aluta	*aluta*	Bedel, 1881	MNCN-AI953	1.08	1.15	0.48	34	37	34	0.025	0.039	0.0149	3.300	1.225	0	0
94	*Limnebius*	*Limnebius*	parvulus-aluta	*lusitanus*	Balfour-Browne, 1979	MNCN-AI899	1.11	1.17	0.36	6	4	5	1.04-1.37	1.03-1.24	0.34-0.39	3.212	1.226	0	0
95	*Limnebius*	*Limnebius*	parvulus-parvulus	*crinifer*	Rey, 1885	IBE-AR55	1.93	1.70	0.70	48	51	31	0.088	0.076	0.0240	5.612	1.320	0	1
96	*Limnebius*	*Limnebius*	parvulus-parvulus	*doderoi*	Gridelli, 1926	MNCN-AI1174	2.05	1.65	0.59	12	28	10	0.132	0.080	0.0475	6.993	1.367	2	1
97	*Limnebius*	*Limnebius*	parvulus-parvulus	*furcatus*	Baudi di Selve, 1872	MNCN-AI987	1.96	1.63	0.57	44	73	27	0.099	0.080	0.0435	6.698	1.364	2	1
98	*Limnebius*	*Limnebius*	parvulus-parvulus	*glabriventris*	Shatrovskiy, 1989		1.92	1.62	0.65	17	23	6	0.070	0.045	0.59-0.68	4.728	1.315	0	0
99	*Limnebius*	*Limnebius*	parvulus-parvulus	*gridellii*	Pretner, 1929		2.07	1.72	0.62	4	29	4	2.02-2.10	0.049	0.59-0.67	6.087	1.321	2	1
100	*Limnebius*	*Limnebius*	parvulus-parvulus	*papposus*	Mulsant, 1844	IBE-RA84	2.07	1.82	0.67	96	97	51	0.093	0.114	0.0543	7.101	1.377	0	1
101	*Limnebius*	*Limnebius*	parvulus-parvulus	*parvulus*	(Herbst, 1797)	MNCN-AI706	1.93	1.70	0.63	42	47	21	0.097	0.076	0.0310	4.651	1.302	0	1

102	*Limnebius*	*Limnebius*	parvulus-parvulus	*reuvenortali*	Jäch, 1993	IBE-AN45	2.02	1.70	0.51	3	10	3	2.00-2.06	0.042	0.49-0.54	5.731	1.313	0	1
103	*Limnebius*	*Limnebius*	parvulus-parvulus	*rubropiceus*	Kuwert, 1890	IBE-AN32	1.93	1.63	0.65	13	74	9	0.073	0.072	0.0200	5.746	1.340	0	1
104	*Limnebius*	*Limnebius*	parvulus-parvulus	*shatrovskiyi*	Jäch, 1993		1.92	1.70	0.75	17	3	7	0.083	1.57-1.75	0.0295	4.229	1.301	0	1
105	*Limnebius*	*Limnebius*	parvulus-parvulus	*stagnalis*	Guillebeau, 1890	MNCN-AI824	1.87	1.63	0.55	78	7	46	0.090	0.061	0.0320	5.442	1.335	0	1
106	*Limnebius*	*Limnebius*	parvulus-setifer	*claviger*	Jäch, 1993		2.31	1.89	0.64	3	2	2	2.27-2.34	1.87-1.9	0.66-0.67	6.695	1.337	0	1
107	*Limnebius*	*Limnebius*	parvulus-setifer	*setifer*	Iablokoff-Khnzorian, 1962		2.13	?	0.71	1	0	1	-	-	-	7.876	1.331	0	1
108	*Limnebius*	*Limnebius*	piceus	*alutaceus*	(Casey, 1886)		1.19	?	0.27	1	0	1	-	-	-	2.642	1.211	0	0
109	*Limnebius*	*Limnebius*	piceus	*angustulus*	(Casey, 1886)		1.12	?	0.29	1	0	1	-	-	-	2.316	1.176	0	0
110	*Limnebius*	*Limnebius*	piceus	*arenicolus*	Perkins, 1980	MNCN-AI466	1.26	1.30	0.47	14	16	14	0.046	0.038	0.0150	2.822	1.216	0	0
111	*Limnebius*	*Limnebius*	piceus	*borealis*	Perkins, 1980		1.18	?	0.53	1	0	1	-	-	-	2.708	1.213	0	0
112	*Limnebius*	*Limnebius*	piceus	*discolor*	Casey, 1900		1.07	?	0.38	1	0	1	-	-	-	2.726	1.197	0	0
113	*Limnebius*	*Limnebius*	piceus	*leechi*	Perkins, 1980		1.12	?	0.35	1	0	1	-	-	-	2.679	1.188	0	0
114	*Limnebius*	*Limnebius*	piceus	*mexicanus*	Perkins, 1980		1.29	?	0.36	1	0	1	-	-	-	2.418	1.210	0	0
115	*Limnebius*	*Limnebius*	piceus	*mitus*	Perkins, 1980		1.27	?	0.27	1	0	1	-	-	-	2.929	1.185	0	0
116	*Limnebius*	*Limnebius*	piceus	*octolaevis*	Perkins, 1980		1.11	?	0.41	1	0	1	-	-	-	2.500	1.209	0	0
117	*Limnebius*	*Limnebius*	piceus	*ozapalachicus*	Perkins, 1980		1.18	?	0.40	1	0	1	-	-	-	2.678	1.199	0	0
118	*Limnebius*	*Limnebius*	piceus	*piceus*	(Horn, 1872)	IBE-RA1120	1.20	1.22	0.45	3	2	3	1.12-1.29	1.19-1.25	0.44-0.46	2.548	1.219	0	0
119	*Limnebius*	*Limnebius*	piceus	*richmondi*	Perkins, 1980		1.30	?	0.53	1	0	1	-	-	-	2.780	1.249	0	0
120	*Limnebius*	*Limnebius*	piceus	*sinuatus*	(Sharp, 1882)	IBE-AR9	1.22	1.29	0.36	1	2	1	-	1.19-1.38	-	2.772	1.195	0	0
121	*Limnebius*	*Limnebius*	piceus	*texanus*	Perkins, 1980		1.20	1.34	0.42	11	11	3	0.065	0.041	0.41-0.44	2.664	1.216	0	0

Evolution of the sexual dimorphism in the beetle genus Limnebius (Hydraenidae) - is sexual selection opportunistic?

Andrey Rudoy & Ignacio Ribera

Table S2

Specimens used in the molecular phylogeny, with country, voucher, species group according to Rudoy et al. (2016) and GenBank accession numbers (from Rudoy & Ribera, 2016).

all	genus	subgenus	species group	species	voucher	country	barcode	COI	16S+tRNA-Leu	18S	28S
1	*Laeliaena*			*Laeliaena sahlbergi*	MNCN-HI19	India	LN995192	LN995232	LN995260	LN995286	-
2	*Limnebius*	*Bilimneus*		*Limnebius sp*	IBE-AF190	India	-	LN995233	LN995261	LN995287	LN995347
3	*Limnebius*	*Bilimneus*		*Limnebius sp*	IBE-AF193	India	-	LN995234	LN995262	LN995288	LN995348
4	*Limnebius*	*Bilimneus*		*Limnebius sp*	MNCN-AI549	Madagascar	-	LN995235	LN995263	LN995289	LN995349
5	*Limnebius*	*Bilimneus*		*Limnebius sp*	MNCN-AI1271	Bhutan	LN995194	LN995236	LN995264	LN995290	LN995350
6	*Limnebius*	*Bilimneus*		*Limnebius sp*	IBE-AR12	Philippines	-	-	LN995265	LN995291	LN995351
7	*Limnebius*	*Bilimneus*		*Limnebius sp*	IBE-RA627	China	LN995195	LN995237	-	-	LN995352
8	*Limnebius*	*Bilimneus*		*Limnebius acupunctus*	MNCN-AI582	Australia	LN995196	LN995238	LN995266	LN995292	LN995353
9	*Limnebius*	*Bilimneus*		*Limnebius atomus*	MNCN-AI1217	Austria	LN995197	HF931174	HF931394	LN995309	LN995354
10	*Limnebius*	*Bilimneus*		*Limnebius dioscoridus*	IBE-RA728	Socotra (Yemen)	LN995198	LN995239	-	LN995310	LN995355
11	*Limnebius*	*Bilimneus*		*Limnebius evanescens*	MNCN-AI915	Morocco	-	HF931241	HF931473	LN995311	LN995356
12	*Limnebius*	*Bilimneus*		*Limnebius extraneus*	IBE-RA89	Spain	LN995199	HF931321	HF931544	LN995312	LN995357
13	*Limnebius*	*Bilimneus*		*Limnebius feuerborni*	IBE-AR11	Bali	LN995200	-	-	LN995313	LN995358
14	*Limnebius*	*Bilimneus*		*Limnebius myrmidon*	MNCN-AI703	Corsica (France)	-	LN995240	LN995267	LN995293	LN995359
15	*Limnebius*	*Bilimneus*		*Limnebius nakanei nakanei*	MNCN-AC30	Japan	LN995208	-	-	LN995314	LN995360
16	*Limnebius*	*Bilimneus*		*Limnebius oblongus*	MNCN-AI942	Morocco		HF931244	HF931479	LN995315	LN995361
17	*Limnebius*	*Bilimneus*		*Limnebius pararabicus*	IBE-RA761	United Arab Emirates	-	-	-	LN995316	LN995362
18	*Limnebius*	*Bilimneus*		*Limnebius perparvulus*	MNCN-AI428	Corsica (France)	-	HF931198	HF931423	LN995317	LN995363
19	*Limnebius*	*Bilimneus*		*Limnebius pollex*	IBE-RA1019	Mauritius	LN995201	-	LN995268	LN995294	LN995364
20	*Limnebius*	*Bilimneus*		*Limnebius wewalkai*	IBE-RA108	Oman	LN995202	LN995241	LN995269	LN995295	LN995365
21	*Limnebius*	*Bilimneus*		*Limnebius endroedyi*	IBE-AN78	South Africa	LN995203	LN995242	LN995270	LN995296	LN995366
22	*Limnebius*	*Limnebius*	gracilipes	*Limnebius cordobanus*	MNCN-AI1024	Spain	HG915298	HF931144	HF931363	LN995318	LN995367
23	*Limnebius*	*Limnebius*	gracilipes	*Limnebius cordobanus*	IBE-PA275	Spain	-	HF931292	HF931527	LN995319	LN995368
24	*Limnebius*	*Limnebius*	gracilipes	*Limnebius fallaciosus*	IBE-RA1121	Greece	-	LN995243	LN995271	-	LN995369
25	*Limnebius*	*Limnebius*	gracilipes	*Limnebius gracilipes*	MNCN-AI1094	Tenerife (Spain)	-	HF931156	HF931375	LN995320	LN995370
26	*Limnebius*	*Limnebius*	nitidus-mundus	*Limnebius murentius*	IBE-AN53	Turkey	-	LN995244	-	-	LN995371
27	*Limnebius*	*Limnebius*	nitidus-nitiduloides	*Limnebius alibeii*	IBE-RA90	Morocco	LN995204	HF931322	HF931545	LN995321	LN995372
28	*Limnebius*	*Limnebius*	nitidus-nitiduloides	*Limnebius crassipes*	IBE-RA68	Crete (Greece)	-	LN995245	LN995272	LN995297	LN995373
29	*Limnebius*	*Limnebius*	nitidus-nitiduloides	*Limnebius fretalis*	MNCN-AI1307	Spain	LN995205	HF931180	HF931401	LN995322	LN995374
30	*Limnebius*	*Limnebius*	nitidus-nitiduloides	*Limnebius hispanicus*	MNCN-AI1309	Spain	HF947987	HF931181	HF931402	LN995323	LN995375
31	*Limnebius*	*Limnebius*	nitidus-nitiduloides	*Limnebius levantinus*	IBE-RA731	Iran	LN995206	-	LN995273	LN995298	LN995376
32	*Limnebius*	*Limnebius*	nitidus-nitiduloides	*Limnebius nitiduloides*	IBE-AR16	Italy	LN995207	LN995246	-	LN995324	-
33	*Limnebius*	*Limnebius*	nitidus-nitiduloides	*Limnebius schoenmanni*	IBE-RA1000	Greece	-	-	LN995274	LN995299	LN995377
34	*Limnebius*	*Limnebius*	nitidus-nitiduloides	*Limnebius simplex*	MNCN-AC39	Sicily (Italy)		HF931110	HF931327	LN995325	LN995378
35	*Limnebius*	*Limnebius*	nitidus-nitiduloides	*Limnebius spinosus*	IBE-AN54	Turkey	LN995209	LN995247	LN995275	LN995300	-
36	*Limnebius*	*Limnebius*	nitidus-nitiduloides	*Limnebius theryi*	MNCN-AI950	Tunisia	LN995210	HF931248	HF931484	LN995326	LN995379
37	*Limnebius*	*Limnebius*	nitidus-nitidus	*Limnebius bacchus*	IBE-PA274	Morocco	-	HF931291	HF931526	LN995327	LN995380
38	*Limnebius*	*Limnebius*	nitidus-nitidus	*Limnebius corfidius*	IBE-AN33	Turkey	LN995211	LN995248	-	-	-
39	*Limnebius*	*Limnebius*	nitidus-nitidus	*Limnebius corybus*	MNCN-AI1280	Turkey		HF931178	HF931399	LN995328	-
40	*Limnebius*	*Limnebius*	nitidus-nitidus	*Limnebius hieronymi*	IBE-AR22	Italy	LN995212	LN995249	LN995276	LN995301	LN995381
41	*Limnebius*	*Limnebius*	nitidus-nitidus	*Limnebius ibericus*	IBE-RA88	Spain	-	LN995250	-	-	LN995382
42	*Limnebius*	*Limnebius*	nitidus-nitidus	*Limnebius kocheri*	IBE-RA91	Morocco	LN995213	HF931323	HF931546	LN995329	LN995383
43	*Limnebius*	*Limnebius*	nitidus-nitidus	*Limnebius minoricensis*	IBE-AR23	Menorca (Spain)	LN995214	HF931265	HF931501	LN995330	LN995384
44	*Limnebius*	*Limnebius*	nitidus-nitidus-cplx	*Limnebius aguilerai*	MNCN-AI910	Morocco	HF947985	HE610230	HF931470	LN995331	LN995385
45	*Limnebius*	*Limnebius*	nitidus-nitidus-cplx	*Limnebius gerhardti*	MNCN-AI891	Spain	HF947986	LN995251	LN995277	LN995302	LN995386
46	*Limnebius*	*Limnebius*	nitidus-nitidus-cplx	*Limnebius hilaris*	MNCN-AI919	Spain	HG915297	HE610232	HF931475	LN995332	LN995387
47	*Limnebius*	*Limnebius*	nitidus-nitidus-cplx	*Limnebius irmelae*	MNCN-AI949	Tunisia		HE610233	HF931483	LN995333	-
48	*Limnebius*	*Limnebius*	nitidus-nitidus-cplx	*Limnebius maurus*	MNCN-AI1317	Mallorca (Spain)	LN995215	LN995252	LN995278	LN995303	LN995388
49	*Limnebius*	*Limnebius*	nitidus-nitidus-cplx	*Limnebius millani*	MNCN-AI920	Spain		HE610235	HF931476	-	LN995389
50	*Limnebius*	*Limnebius*	nitidus-nitidus-cplx	*Limnebius monfortei*	IBE-AF199	Spain	-	HE610236	HF931341	-	LN995390
51	*Limnebius*	*Limnebius*	nitidus-nitidus-cplx	*Limnebius montanus*	MNCN-AI936	Spain	-	HE610237	HF931478	LN995334	LN995391
52	*Limnebius*	*Limnebius*	nitidus-nitidus-cplx	*Limnebius nitidus*	MNCN-AI377	Spain	LN995216	HE610238	HF931415	LN995335	LN995392
53	*Limnebius*	*Limnebius*	nitidus-nitidus-cplx	*Limnebius ordunyai*	MNCN-AI848	Spain	HF947989	LN995253	LN995279	-	-
54	*Limnebius*	*Limnebius*	nitidus-punctatus	*Limnebius ignarus*	MNCN-AI993	Spain	HF947988	HF931262	HF931498	-	LN995393
55	*Limnebius*	*Limnebius*	nitidus-punctatus	*Limnebius mucronatus*	MNCN-AI704	Corsica (France)	LN995217	HF931223	HF931452	LN995336	LN995394
56	*Limnebius*	*Limnebius*	nitidus-punctatus	*Limnebius pilicauda*	IBE-AR60	Morocco	LN995218	LN995254	LN995280	LN995304	LN995395
57	*Limnebius*	*Limnebius*	nitidus-punctatus	*Limnebius zaerensis*	MNCN-AC14	Morocco	-	EU365868	+EU365866+	HE970937	HE970984
58	*Limnebius*	*Limnebius*	nitidus-truncatellus	*Limnebius mesatlanticus*	MNCN-AI913	Morocco	-	HF931239	HF931471	LN995337	LN995396
59	*Limnebius*	*Limnebius*	nitidus-truncatellus	*Limnebius truncatellus*	MNCN-AI451	Spain	LN995219	HF931201	HF931426	LN995338	LN995397
60	*Limnebius*	*Limnebius*	parvulus-aluta	*Limnebius aluta*	MNCN-AI953	England (U.K.)	LN995220	HF931249	HF931485	LN995339	LN995398
61	*Limnebius*	*Limnebius*	parvulus-aluta	*Limnebius lusitanus*	MNCN-AI899	Spain	HG915296	HF931238	HF931468	LN995340	LN995399
62	*Limnebius*	*Limnebius*	parvulus-parvulus	*Limnebius crinifer*	IBE-AR55	Sweden	LN995221	LN995255	LN995281	LN995305	LN995400
63	*Limnebius*	*Limnebius*	parvulus-parvulus	*Limnebius doderoi*	MNCN-AI1174	Corsica (France)	LN995222	HF931170	HF931389	LN995341	LN995401
64	*Limnebius*	*Limnebius*	parvulus-parvulus	*Limnebius furcatus*	MNCN-AI987	Spain	LN995223	LN995256	LN995282	-	-
65	*Limnebius*	*Limnebius*	parvulus-parvulus	*Limnebius papposus*	IBE-RA84	Montenegro	LN995224	LN995257	LN995283	LN995306	LN995402
66	*Limnebius*	*Limnebius*	parvulus-parvulus	*Limnebius parvulus*	MNCN-AI706	Poland	LN995225	HF931224	HF931453	LN995342	LN995403
67	*Limnebius*	*Limnebius*	parvulus-parvulus	*Limnebius reuvenortali*	IBE-AN45	Turkey	LN995226	LN995258	LN995284	LN995307	LN995404
68	*Limnebius*	*Limnebius*	parvulus-parvulus	*Limnebius rubropiceus*	IBE-AN32	Turkey	LN995227	LN995259	LN995285	LN995308	LN995405
69	*Limnebius*	*Limnebius*	parvulus-parvulus	*Limnebius stagnalis*	MNCN-AI824	Turkey	LN995228	HF931230	HF931459	LN995343	LN995406
70	*Limnebius*	*Limnebius*	piceus	*Limnebius arenicolus*	MNCN-AI466	California (U.S.)	LN995229	HF931204	HF931429	LN995344	LN995407
71	*Limnebius*	*Limnebius*	piceus	*Limnebius piceus*	IBE-RA1120	California (U.S.)	LN995230	-	-	LN995345	LN995408
72	*Limnebius*	*Limnebius*	piceus	*Limnebius sinuatus*	IBE-AR9	Arizona (U.S.)	LN995231	-	-	LN995346	LN995409

Evolution of the sexual dimorphism in the beetle genus Limnebius (Hydraenidae) - is sexual selection opportunistic?

Andrey Rudoy & Ignacio Ribera

Table S3

Reconstructed values of the measured variables in all branches of the phylogeny and estimated measurements of SSD.

See Figure S3 for the location of the branch numbers in the phylogeny. The raw values of male and female body size of the initial and final nodes of each branch are given, together with the amount of phenotypic change and the change measured in darwins (x10

In pink, composite branch after deleting terminals with missing males. In yellow, estimated values of lf.

				branch measures								SSD measures						qualitative me [0.975+/-0.016					
				initial	final	initial	final	phenotypic change		darwins x10^6		rSSD	absolute dif	SSD change	darwins cha	>5% change		phenotypic cha			>5% change		
branch	compound	terminal	subgenus	lfi	lff	lmi	lmf	p(lf)	p(lm)	d(lf)	d(lm)	lm/lf	ABS(p(lm))-	(ABS(lmf-lff	d(lm)-d(lf)	lf	lm	lf	lm	SSD (	lf	lm	SSD (
1		sahlbergi	Laeliaena	1.250	1.247	1.270	1.420	-0.003	0.150	0.000	0.003	1.139	0.147	0.154	0.003	0.997	1.118	-	+	+	=	+	+
2			stem Limnebius	1.250	1.251	1.270	1.249	0.001	-0.021	0.000	0.003	0.998	0.020	-0.017	0.003	1.001	0.984	+	-	-	=	=	-
3			stem Bilimneus	1.251	1.101	1.249	0.994	-0.150	-0.256	0.012	0.021	0.902	0.106	0.106	0.009	0.880	0.795	-	-	+	-	-	+
4			stem s.str.	1.251	1.392	1.249	1.438	0.140	0.189	0.011	0.014	1.033	0.049	0.044	0.003	1.112	1.151	+	+	+	+	+	+
5			Bilimneus	1.101	1.096	0.994	0.968	-0.005	-0.026	0.002	0.013	0.883	0.021	0.021	0.011	0.996	0.974	-	-	+	=	-	+
6			Bilimneus	1.101	1.048	0.994	0.945	-0.053	-0.049	0.012	0.012	0.902	-0.005	-0.005	0.000	0.951	0.951	-	-	-	-	-	=
8			Bilimneus	1.096	1.093	0.968	0.947	-0.003	-0.021	0.001	0.011	0.866	0.018	0.018	0.010	0.997	0.979	-	-	+	=	=	+
10	7+10	pollex	Bilimneus	1.096	1.100	0.968	0.930	0.003	-0.038	0.000	0.002	0.846	0.034	0.041	0.002	1.003	0.961	+	-	+	=	-	+
11		sp India	Bilimneus	1.093	1.092	0.947	0.830	-0.001	-0.117	0.000	0.009	0.760	0.116	0.116	0.009	0.999	0.877	-	-	+	=	-	+
12			Bilimneus	1.093	1.084	0.947	0.937	-0.009	-0.010	0.002	0.003	0.864	0.001	0.001	0.001	0.991	0.989	-	-	+	=	=	=
13		feuerborni	Bilimneus	1.084	1.084	0.937	0.830	0.000	-0.107	0.000	0.011	0.766	0.106	0.107	0.011	1.000	0.886	+	-	+	=	-	+
15	14+15	atomus	Bilimneus	1.084	1.060	0.937	0.990	-0.024	0.053	0.002	0.005	0.934	0.029	-0.077	0.003	0.978	1.057	-	+	-	=	+	-
17			Bilimneus	1.048	1.035	0.945	0.904	-0.013	-0.041	0.002	0.008	0.873	0.028	0.028	0.006	0.988	0.956	-	-	+	=	-	+
18			Bilimneus	1.048	1.019	0.945	0.933	-0.028	-0.012	0.011	0.005	0.915	-0.016	-0.016	-0.006	0.973	0.987	-	-	-	-	=	=
19		endroedyi	Bilimneus	1.035	1.080	0.904	0.860	0.045	-0.044	0.005	0.006	0.796	-0.001	0.089	0.001	1.043	0.952	+	-	+	+	-	+
20			Bilimneus	1.035	1.022	0.904	0.902	-0.013	-0.002	0.007	0.001	0.882	-0.011	-0.011	-0.006	0.987	0.998	-	-	-	=	=	=
21		evanescens	Bilimneus	1.022	1.010	0.902	0.880	-0.012	-0.022	0.002	0.004	0.871	0.009	0.009	0.002	0.988	0.976	-	-	+	=	=	=
22			Bilimneus	1.022	1.007	0.902	0.904	-0.015	0.003	0.006	0.001	0.898	-0.012	-0.018	-0.005	0.986	1.003	-	+	-	=	=	-
23		extraneus	Bilimneus	1.007	0.950	0.904	0.840	-0.057	-0.064	0.014	0.018	0.884	0.007	0.007	0.004	0.943	0.929	-	-	+	-	-	=
24			Bilimneus	1.007	1.028	0.904	0.944	0.021	0.039	0.009	0.018	0.918	0.019	-0.019	0.009	1.021	1.044	+	+	-	=	+	-
25		perparvulus	Bilimneus	1.028	1.000	0.944	0.910	-0.028	-0.034	0.016	0.021	0.910	0.006	0.006	0.005	0.973	0.964	-	-	+	-	-	=
26		oblongus	Bilimneus	1.028	1.080	0.944	1.010	0.052	0.066	0.029	0.039	0.935	0.014	-0.014	0.011	1.051	1.070	+	+	-	+	+	=
27			Bilimneus	1.019	0.991	0.933	0.913	-0.028	-0.020	0.009	0.007	0.921	-0.008	-0.008	-0.002	0.972	0.978	-	-	-	-	=	=
28			Bilimneus	1.019	1.019	0.933	0.941	0.000	0.008	0.000	0.005	0.923	0.007	-0.008	0.005	1.000	1.008	-	+	-	=	=	=
29		myrmidon	Bilimneus	0.991	0.970	0.913	0.890	-0.021	-0.023	0.002	0.003	0.918	0.001	0.001	0.000	0.979	0.975	-	-	+	=	=	=
30			Bilimneus	0.991	0.977	0.913	0.906	-0.014	-0.007	0.006	0.003	0.927	-0.007	-0.007	-0.003	0.986	0.992	-	-	-	=	=	=
31		wewalkai	Bilimneus	0.977	0.910	0.906	0.830	-0.067	-0.076	0.011	0.014	0.912	0.009	0.009	0.003	0.931	0.916	-	-	+	-	-	=
32			Bilimneus	0.977	0.992	0.906	0.936	0.015	0.031	0.003	0.007	0.944	0.016	-0.016	0.004	1.015	1.034	+	+	-	=	+	=
33		pararabicus	Bilimneus	0.992	1.000	0.936	0.820	0.008	-0.116	0.004	0.069	0.820	0.108	0.124	0.065	1.008	0.876	+	-	+	=	-	+
34		dioscoridus	Bilimneus	0.992	0.993	0.936	1.070	0.001	0.134	0.000	0.069	1.078	0.133	0.022	0.069	1.001	1.143	+	+	+	=	+	+
37	35+37	sp Buthan	Bilimneus	1.019	1.060	0.941	1.040	0.041	0.099	0.004	0.010	0.981	0.058	-0.058	0.006	1.040	1.106	+	+	-	+	+	-
41	36+40+41	nakanei	Bilimneus	1.019	0.980	0.941	0.860	-0.039	-0.081	0.004	0.009	0.878	0.042	0.042	0.005	0.962	0.914	-	-	+	-	-	+
43			Limnebius s.str.	1.392	1.425	1.438	1.510	0.033	0.072	0.006	0.013	1.060	0.039	0.039	0.006	1.024	1.050	+	+	+	=	+	+
44			Limnebius s.str.	1.392	1.391	1.438	1.429	-0.001	-0.009	0.000	0.005	1.028	0.008	-0.008	0.004	0.999	0.994	-	-	-	=	=	=
45			Limnebius s.str.	1.425	1.278	1.510	1.280	-0.147	-0.229	0.018	0.027	1.001	0.083	-0.083	0.009	0.897	0.848	-	-	-	-	-	-
46			Limnebius s.str.	1.425	1.542	1.510	1.710	0.117	0.200	0.024	0.038	1.109	0.083	0.083	0.014	1.082	1.132	+	+	+	+	+	+
47		aluta	Limnebius s.str.	1.278	1.150	1.280	1.080	-0.128	-0.200	0.011	0.018	0.939	0.072	0.068	0.007	0.900	0.844	-	-	+	-	-	+
48		lusitanus	Limnebius s.str.	1.278	1.170	1.280	1.110	-0.108	-0.170	0.009	0.015	0.949	0.062	0.058	0.006	0.915	0.867	-	-	+	-	-	+
49			Limnebius s.str.	1.542	1.665	1.710	1.871	0.123	0.161	0.014	0.016	1.124	0.038	0.038	0.002	1.080	1.094	+	+	+	+	+	+
50			Limnebius s.str.	1.542	1.594	1.710	1.835	0.052	0.126	0.009	0.019	1.151	0.073	0.073	0.010	1.034	1.073	+	+	+	+	+	+
51		parvulus	Limnebius s.str.	1.665	1.700	1.871	1.930	0.035	0.059	0.003	0.005	1.135	0.024	0.024	0.002	1.021	1.032	+	+	+	=	+	+

52		Limnebius s.str.	1.665	1.728	1.871	1.945	0.063	0.074	0.011	0.011	1.126	0.011	0.011	0.000	1.038	1.039	+	+	+	+	+	=
53	rubropiceus	Limnebius s.str.	1.728	1.740	1.945	1.930	0.012	-0.015	0.002	0.002	1.109	0.003	-0.027	0.000	1.007	0.992	+	-	-	=	=	-
54		Limnebius s.str.	1.728	1.743	1.945	1.972	0.015	0.027	0.008	0.013	1.131	0.011	0.011	0.005	1.009	1.014	+	+	+	=	=	=
55	crinifer	Limnebius s.str.	1.743	1.700	1.972	1.930	-0.043	-0.042	0.012	0.010	1.135	-0.002	0.002	-0.002	0.975	0.979	-	-	+	=	=	=
56	papposus	Limnebius s.str.	1.743	1.820	1.972	2.070	0.077	0.098	0.020	0.023	1.137	0.022	0.022	0.003	1.044	1.050	+	+	+	+	+	+
57		Limnebius s.str.	1.594	1.637	1.835	1.996	0.043	0.161	0.003	0.011	1.219	0.118	0.118	0.007	1.027	1.088	+	+	+	+	+	+
58		Limnebius s.str.	1.594	1.662	1.835	1.940	0.068	0.105	0.005	0.007	1.167	0.037	0.037	0.002	1.043	1.057	+	+	+	+	+	+
59	doderoi	Limnebius s.str.	1.637	1.650	1.996	2.050	0.013	0.054	0.010	0.034	1.242	0.041	0.041	0.024	1.008	1.027	+	+	+	=	+	+
60	furcatus	Limnebius s.str.	1.637	1.630	1.996	1.960	-0.007	-0.036	0.006	0.023	1.202	0.029	-0.029	0.017	0.996	0.982	-	-	-	=	=	-
61	stagnalis	Limnebius s.str.	1.662	1.630	1.940	1.870	-0.032	-0.070	0.031	0.058	1.147	0.038	-0.038	0.027	0.981	0.964	-	-	-	=	-	-
62	reuvenortali	Limnebius s.str.	1.662	1.700	1.940	2.020	0.038	0.080	0.035	0.063	1.188	0.042	0.042	0.028	1.023	1.041	+	+	+	=	+	+
63		Limnebius s.str.	1.391	1.338	1.429	1.347	-0.053	-0.082	0.014	0.021	1.007	0.029	-0.029	0.007	0.962	0.943	-	-	-	-	-	-
64		Limnebius s.str.	1.391	1.506	1.429	1.579	0.115	0.150	0.018	0.022	1.049	0.035	0.035	0.005	1.083	1.105	+	+	+	+	+	+
65		Limnebius s.str.	1.338	1.273	1.347	1.207	-0.065	-0.140	0.004	0.009	0.948	0.076	0.056	0.005	0.952	0.896	-	-	+	-	-	+
66		Limnebius s.str.	1.338	1.274	1.347	1.253	-0.064	-0.095	0.010	0.015	0.984	0.031	0.011	0.005	0.952	0.930	-	-	+	-	-	=
67	piceus	Limnebius s.str.	1.273	1.220	1.207	1.120	-0.053	-0.087	0.017	0.030	0.918	0.034	0.034	0.013	0.958	0.928	-	-	+	-	-	+
68		Limnebius s.str.	1.273	1.283	1.207	1.222	0.010	0.015	0.009	0.014	0.953	0.005	-0.005	0.005	1.008	1.013	+	+	-	=	=	=
69	sinuatus	Limnebius s.str.	1.283	1.290	1.222	1.220	0.007	-0.002	0.003	0.001	0.946	-0.005	0.009	-0.002	1.005	0.998	+	-	+	=	=	=
70	arenicolus	Limnebius s.str.	1.283	1.300	1.222	1.260	0.017	0.038	0.008	0.020	0.969	0.021	-0.021	0.011	1.013	1.031	+	+	-	=	+	-
71		Limnebius s.str.	1.274	1.292	1.253	1.266	0.019	0.013	0.009	0.007	0.980	-0.005	0.005	-0.003	1.015	1.010	+	+	+	=	=	=
72 72+75	cordobanus	Limnebius s.str.	1.274	1.010	1.253	0.920	-0.264	-0.333	0.022	0.030	0.911	0.069	0.069	0.007	0.793	0.734	-	-	+	-	-	+
73	fallaciosus	Limnebius s.str.	1.292	1.400	1.266	1.310	0.108	0.044	0.009	0.004	0.936	-0.064	0.064	-0.005	1.084	1.035	+	+	+	+	+	+
74	gracilipes	Limnebius s.str.	1.292	1.281	1.266	1.320	-0.011	0.054	0.001	0.005	1.030	0.044	0.012	0.004	0.992	1.043	-	+	+	=	+	=
77		Limnebius s.str.	1.506	1.868	1.579	2.052	0.362	0.473	0.030	0.036	1.099	0.111	0.111	0.006	1.240	1.299	+	+	+	+	+	+
78		Limnebius s.str.	1.506	1.517	1.579	1.595	0.011	0.015	0.008	0.010	1.051	0.004	0.004	0.002	1.007	1.010	+	+	+	=	=	=
79		Limnebius s.str.	1.868	1.933	2.052	2.144	0.065	0.092	0.011	0.014	1.109	0.027	0.027	0.003	1.035	1.045	+	+	+	+	+	+
80		Limnebius s.str.	1.868	1.893	2.052	2.081	0.026	0.029	0.017	0.017	1.099	0.003	0.003	0.000	1.014	1.014	+	+	+	=	=	=
81	hispanicus	Limnebius s.str.	1.933	1.900	2.144	2.060	-0.033	-0.084	0.005	0.013	1.084	0.051	-0.051	0.007	0.983	0.961	-	-	-	=	-	-
82		Limnebius s.str.	1.933	1.971	2.144	2.215	0.038	0.071	0.014	0.023	1.124	0.033	0.033	0.009	1.020	1.033	+	+	+	=	+	+
83	theryi	Limnebius s.str.	1.971	1.920	2.215	2.240	-0.051	0.025	0.015	0.006	1.167	-0.026	0.076	-0.009	0.974	1.011	-	+	+	-	=	+
84		Limnebius s.str.	1.971	2.037	2.215	2.262	0.066	0.047	0.028	0.018	1.111	-0.019	-0.019	-0.010	1.033	1.021	+	+	-	+	=	-
85	fretalis	Limnebius s.str.	2.037	2.120	2.262	2.390	0.083	0.128	0.069	0.095	1.127	0.045	0.045	0.026	1.041	1.056	+	+	+	+	+	+
86	alibeii	Limnebius s.str.	2.037	1.990	2.262	2.160	-0.047	-0.102	0.040	0.080	1.085	0.055	-0.055	0.040	0.977	0.955	-	-	-	=	-	-
87		Limnebius s.str.	1.893	1.830	2.081	2.008	-0.063	-0.073	0.010	0.010	1.097	0.010	-0.010	0.000	0.967	0.965	-	-	-	-	-	=
88		Limnebius s.str.	1.893	1.944	2.081	2.140	0.050	0.059	0.025	0.026	1.101	0.008	0.008	0.001	1.027	1.028	+	+	+	+	+	=
89	spinosus	Limnebius s.str.	1.830	1.830	2.008	1.980	0.000	-0.028	0.000	0.006	1.082	0.028	-0.028	0.006	1.000	0.986	+	-	-	=	=	-
90	levantinus	Limnebius s.str.	1.830	1.800	2.008	2.000	-0.030	-0.008	0.008	0.002	1.111	-0.022	0.022	-0.006	0.984	0.996	-	-	+	=	=	+
91		Limnebius s.str.	1.944	2.014	2.140	2.170	0.070	0.030	0.025	0.010	1.077	-0.040	-0.040	-0.015	1.036	1.014	+	+	-	+	=	-
92		Limnebius s.str.	1.944	1.931	2.140	2.198	-0.012	0.058	0.003	0.012	1.138	0.046	0.071	0.009	0.994	1.027	-	+	+	=	+	+
93	simplex	Limnebius s.str.	2.014	1.960	2.170	1.960	-0.054	-0.210	0.009	0.034	1.000	0.156	-0.156	0.025	0.973	0.903	-	-	-	-	-	-
94	nitiduloides	Limnebius s.str.	2.014	2.220	2.170	2.450	0.206	0.280	0.032	0.040	1.104	0.074	0.074	0.008	1.102	1.129	+	+	+	+	+	+
95	crassipes	Limnebius s.str.	1.931	1.920	2.198	2.330	-0.011	0.132	0.003	0.026	1.214	0.121	0.143	0.024	0.994	1.060	-	+	+	=	+	+
96	schoenmanni	Limnebius s.str.	1.931	1.933	2.198	2.130	0.001	-0.068	0.000	0.014	1.102	0.066	-0.069	0.014	1.001	0.969	+	-	-	=	-	-
97		Limnebius s.str.	1.517	1.291	1.595	1.325	-0.225	-0.270	0.021	0.024	1.026	0.044	-0.044	0.003	0.851	0.831	-	-	-	-	-	-
98		Limnebius s.str.	1.517	1.522	1.595	1.600	0.005	0.005	0.003	0.003	1.051	0.000	0.000	0.000	1.004	1.003	+	+	=	=	=	=
99	mucronatus	Limnebius s.str.	1.291	1.290	1.325	1.180	-0.001	-0.145	0.000	0.024	0.915	0.144	0.076	0.023	0.999	0.891	-	-	+	=	-	+
100		Limnebius s.str.	1.291	1.214	1.325	1.314	-0.077	-0.011	0.021	0.003	1.082	-0.066	0.066	-0.018	0.940	0.992	-	-	+	-	=	+
101	pilicauda	Limnebius s.str.	1.214	1.270	1.314	1.510	0.056	0.196	0.022	0.069	1.189	0.140	0.140	0.046	1.046	1.149	+	+	+	+	+	+
102		Limnebius s.str.	1.214	1.192	1.314	1.287	-0.023	-0.027	0.048	0.053	1.080	0.004	-0.004	0.004	0.981	0.980	-	-	-	=	=	=
103	zaerensis	Limnebius s.str.	1.192	1.207	1.287	1.310	0.015	0.023	0.008	0.011	1.085	0.007	0.007	0.003	1.013	1.018	+	+	+	=	=	=
104	ignarus	Limnebius s.str.	1.192	1.100	1.287	1.110	-0.092	-0.177	0.049	0.091	1.009	0.086	-0.086	0.042	0.923	0.862	-	-	-	-	-	-
105		Limnebius s.str.	1.522	1.862	1.600	2.086	0.340	0.486	0.028	0.036	1.120	0.146	0.146	0.009	1.223	1.304	+	+	+	+	+	+

106		Limnebius s.str.	1.522	1.332	1.600	1.316	-0.190	-0.284	0.029	0.043	0.987	0.094	-0.061	0.014	0.875	0.822	-	-	-	-	-	-
107	truncatellus	Limnebius s.str.	1.862	1.980	2.086	2.380	0.118	0.294	0.014	0.031	1.202	0.177	0.177	0.017	1.063	1.141	+	+	+	+	+	+
108	mesatlanticus	Limnebius s.str.	1.862	1.910	2.086	2.040	0.048	-0.046	0.006	0.005	1.068	-0.002	-0.093	-0.001	1.026	0.978	+	-	-	+	=	-
109		Limnebius s.str.	1.332	1.289	1.316	1.261	-0.044	-0.055	0.024	0.031	0.978	0.011	0.011	0.007	0.967	0.958	-	-	+	-	-	=
110		Limnebius s.str.	1.332	1.332	1.316	1.303	0.000	-0.013	0.000	0.011	0.978	0.013	0.013	0.011	1.000	0.990	-	-	+	=	=	=
111	murentius	Limnebius s.str.	1.332	1.150	1.261	1.090	-0.182	-0.171	0.026	0.026	0.948	-0.012	-0.012	0.000	0.863	0.865	-	-	-	-	-	=
112		Limnebius s.str.	1.332	1.288	1.261	1.261	-0.044	0.000	0.081	0.001	0.979	-0.044	-0.045	-0.080	0.967	1.000	-	+	-	-	=	-
113	hieronymi	Limnebius s.str.	1.288	1.280	1.261	1.280	-0.008	0.019	0.001	0.003	1.000	0.011	-0.027	0.002	0.994	1.015	-	+	-	=	=	-
114		Limnebius s.str.	1.288	1.245	1.261	1.220	-0.043	-0.041	0.021	0.021	0.980	-0.002	-0.002	0.000	0.967	0.967	-	-	-	-	-	=
115	corfidius	Limnebius s.str.	1.245	1.190	1.220	1.140	-0.055	-0.080	0.012	0.019	0.958	0.025	0.025	0.006	0.956	0.934	-	-	+	-	-	+
116	corybus	Limnebius s.str.	1.245	1.250	1.220	1.220	0.005	0.000	0.001	0.000	0.976	-0.005	0.005	-0.001	1.004	1.000	+	=	+	=	=	=
117	minoricensis	Limnebius s.str.	1.332	1.400	1.303	1.240	0.068	-0.063	0.008	0.008	0.886	-0.005	0.131	0.000	1.051	0.952	+	-	+	+	-	+
118		Limnebius s.str.	1.332	1.327	1.303	1.301	-0.005	-0.002	0.007	0.003	0.980	-0.003	-0.003	-0.004	0.996	0.999	-	-	-	=	=	=
119	bacchus	Limnebius s.str.	1.327	1.270	1.301	1.260	-0.057	-0.041	0.008	0.006	0.992	-0.017	-0.017	-0.002	0.957	0.969	-	-	-	-	-	-
120		Limnebius s.str.	1.327	1.331	1.301	1.312	0.004	0.012	0.005	0.017	0.986	0.008	-0.008	0.012	1.003	1.009	+	+	-	=	=	=
121	kocheri	Limnebius s.str.	1.331	1.350	1.312	1.330	0.019	0.018	0.003	0.003	0.985	-0.002	0.002	0.000	1.014	1.013	+	+	+	=	=	=
122		Limnebius s.str.	1.331	1.329	1.312	1.308	-0.002	-0.004	0.002	0.004	0.984	0.002	0.002	0.003	0.999	0.997	-	-	+	=	=	=
123	ibericus	Limnebius s.str.	1.329	1.260	1.308	1.210	-0.069	-0.098	0.012	0.018	0.960	0.029	0.029	0.006	0.948	0.925	-	-	+	-	-	+
124		Limnebius s.str.	1.329	1.376	1.308	1.385	0.047	0.076	0.011	0.018	1.006	0.029	-0.013	0.007	1.035	1.058	+	+	-	+	+	=
125		Limnebius s.str.	1.376	1.373	1.385	1.383	-0.003	-0.002	0.005	0.003	1.007	-0.001	0.001	-0.001	0.998	0.998	-	-	+	=	=	=
126		Limnebius s.str.	1.376	1.381	1.385	1.394	0.004	0.009	0.008	0.018	1.009	0.005	0.005	0.009	1.003	1.006	+	+	+	=	=	=
127	montanus	Limnebius s.str.	1.373	1.360	1.383	1.360	-0.013	-0.023	0.013	0.022	1.000	0.009	-0.009	0.009	0.990	0.984	-	-	-	=	=	=
128	nitidus	Limnebius s.str.	1.373	1.380	1.383	1.400	0.007	0.017	0.007	0.017	1.014	0.011	0.011	0.010	1.005	1.013	+	+	+	=	=	=
129	irmelae	Limnebius s.str.	1.381	1.410	1.394	1.430	0.029	0.036	0.023	0.028	1.014	0.007	0.007	0.005	1.021	1.026	+	+	+	=	+	=
130		Limnebius s.str.	1.381	1.381	1.394	1.390	0.001	-0.004	0.002	0.008	1.006	0.003	-0.004	0.006	1.001	0.997	+	-	-	=	=	=
131		Limnebius s.str.	1.381	1.372	1.390	1.389	-0.009	-0.001	0.034	0.004	1.012	-0.008	0.008	-0.029	0.993	0.999	-	-	+	=	=	=
132		Limnebius s.str.	1.381	1.385	1.390	1.390	0.003	0.000	0.024	0.002	1.004	-0.003	-0.004	-0.022	1.002	1.000	+	-	-	=	=	=
133	maurus	Limnebius s.str.	1.372	1.410	1.389	1.440	0.038	0.051	0.072	0.096	1.021	0.013	0.013	0.024	1.028	1.037	+	+	+	+	+	=
134		Limnebius s.str.	1.372	1.361	1.389	1.380	-0.012	-0.009	0.081	0.065	1.014	-0.002	0.002	-0.016	0.992	0.993	-	-	+	=	=	=
135	gerhardti	Limnebius s.str.	1.361	1.380	1.380	1.420	0.019	0.041	0.051	0.105	1.029	0.021	0.021	0.054	1.014	1.029	+	+	+	=	+	+
136	ordunyai	Limnebius s.str.	1.361	1.300	1.380	1.300	-0.061	-0.079	0.166	0.216	1.000	0.019	-0.019	0.050	0.955	0.942	-	-	-	-	-	-
137	monfortei	Limnebius s.str.	1.385	1.450	1.390	1.440	0.065	0.050	0.097	0.075	0.993	-0.015	0.005	-0.022	1.047	1.036	+	+	+	+	+	=
138		Limnebius s.str.	1.385	1.374	1.390	1.377	-0.011	-0.013	0.062	0.075	1.002	0.002	-0.002	0.012	0.992	0.991	-	-	-	=	=	=
139	hilaris	Limnebius s.str.	1.374	1.330	1.377	1.350	-0.044	-0.027	0.093	0.056	1.015	-0.017	0.017	-0.037	0.968	0.981	-	-	+	-	=	+
140		Limnebius s.str.	1.374	1.378	1.377	1.374	0.004	-0.003	0.034	0.022	0.997	-0.002	0.001	-0.012	1.003	0.998	+	-	+	=	=	=
141	millani	Limnebius s.str.	1.378	1.390	1.374	1.360	0.012	-0.014	0.033	0.039	0.978	0.002	0.026	0.006	1.009	0.990	+	-	+	=	=	+
142	aguilerai	Limnebius s.str.	1.378	1.370	1.374	1.380	-0.008	0.006	0.023	0.017	1.007	-0.002	0.006	-0.006	0.994	1.004	-	+	+	=	=	=

Figure S1. Aedeagus of *L. gerhardti*, with A) maximum length (*lg*); B) perimeter, outlined by hand and measured in ImageJ; and C) log-log plot of the regression between the scale of the box and the number of boxes necessary to cover the image, obtained with the Fractal Dimension Estimator (see Methods). The absolute value of the slope of the regression line is the fractal dimension of the image (in this case, the outline of the aedeagus).

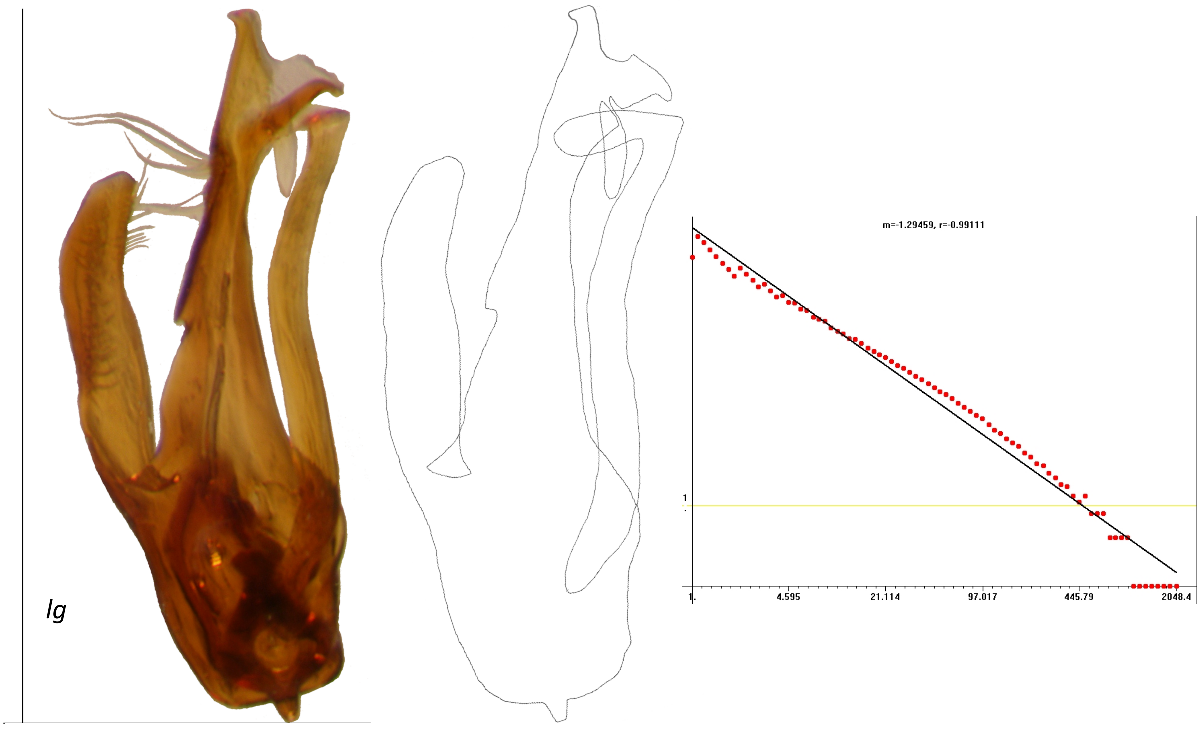

A B C

Figure S2. Male secondary sexual characters in the genus *Limnebius*. A & B) Abdominal modifications in ventral and lateral views (A, *L. fretalis*; B, *L. furcatus*); C to E) modification of the hind tibiae of C, *L. mesatlanticus*; D, *L. truncatellus*, the last two with a widening of the distal part; E, *L. fretalis*, with a row of strong setae; F) typical unmodified tibiae of female species of *Limnebius* (*L. fretalis*).

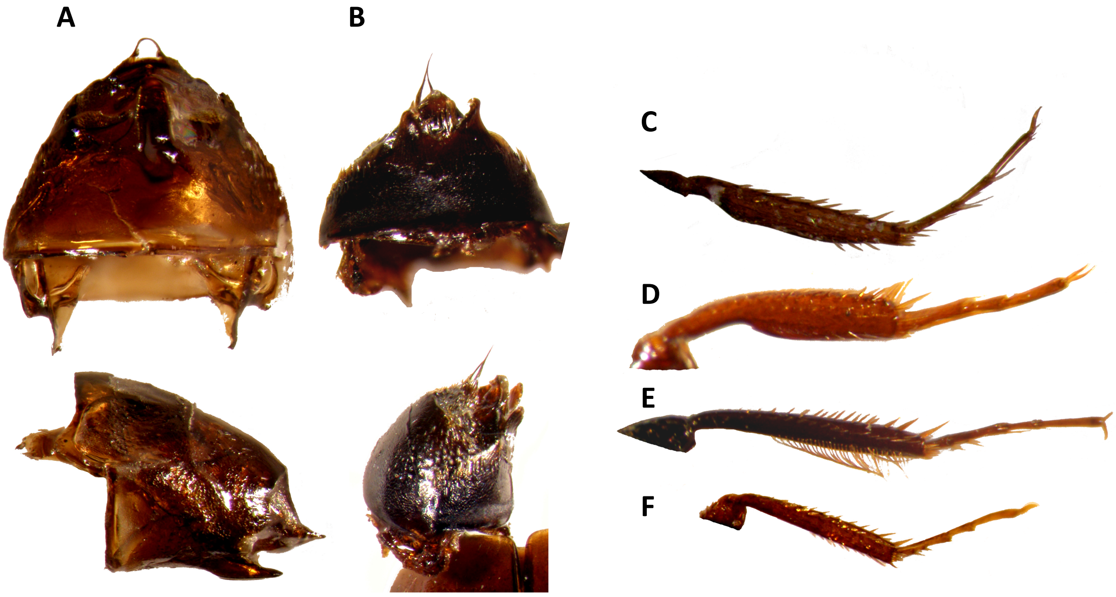

Figure S3. Estimated relationships of the species of *Limnebius* for which no molecular data were available (species without voucher numbers), based mostly on the general shape and structure of the male genitalia (from Rudoy et al., 2016).

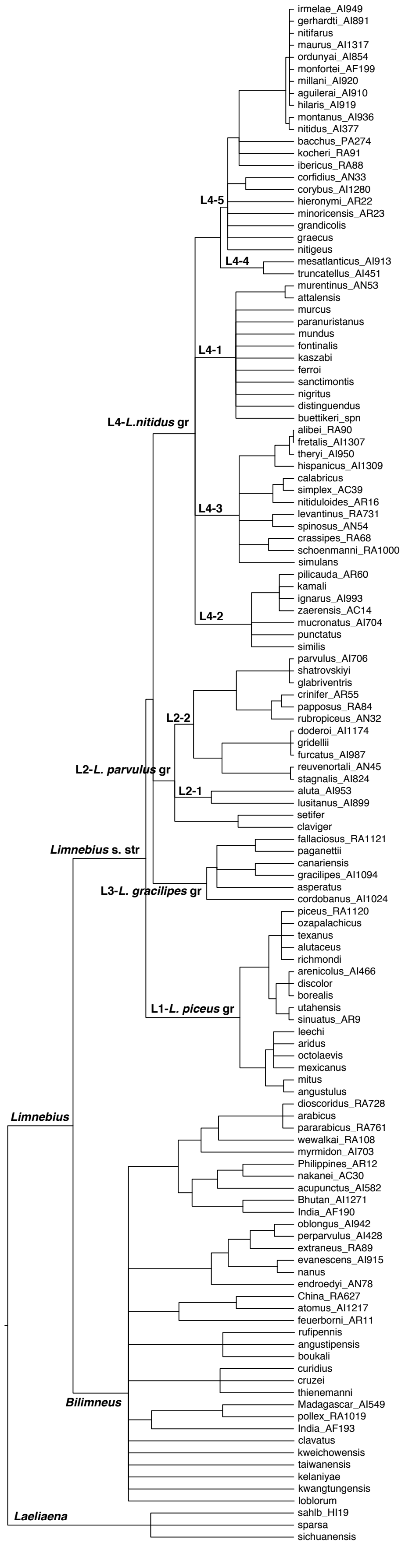

Figure S4. Phylogeny of *Limnebius*, with node support (posterior probability as estimated in BEAST) and the branch numbers used in Table S3 (from Rudoy & Ribera, 2016). See Tables S1 and S2 for details on the species.

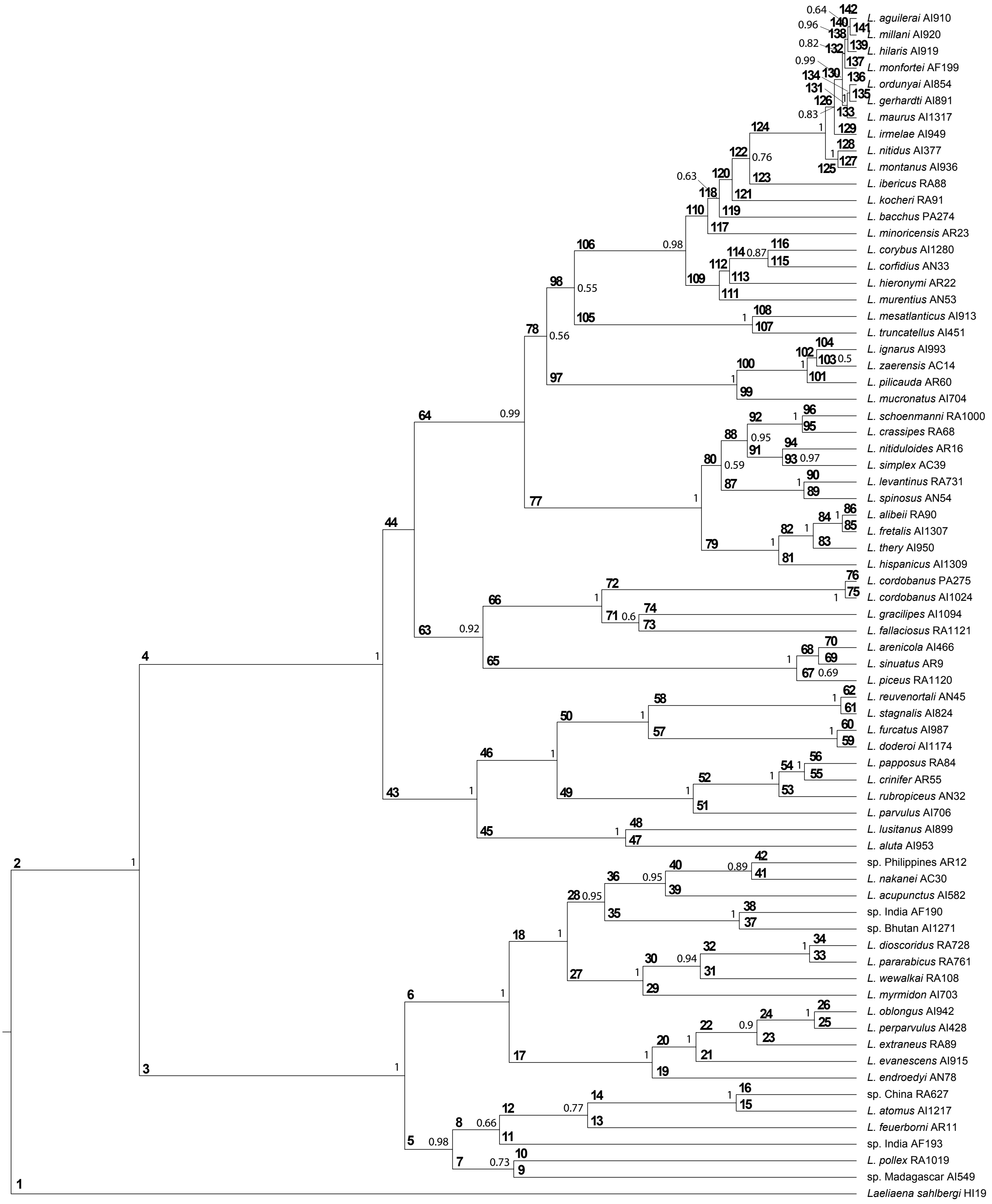

Figure S5. Evolutionary trajectories of de SSD in all the species of *Limnebius* included in the molecular phylogeny. In the y-axis, reconstructed values of male (red) and female (blue) body sizes (mm); in the x-axis, nodes in the reconstructed evolution of the species. Distances in the x-axis are proportional to time (MY, note that the scale is reversed, i.e. the root of the *Limnebiini* tree is dated with time 0). The trajectory represented in F (*L. nitidus* complex) is the same for all the species in this complex, due to the short terminal branches and the uncertain relationships among the species.

Bilimneus

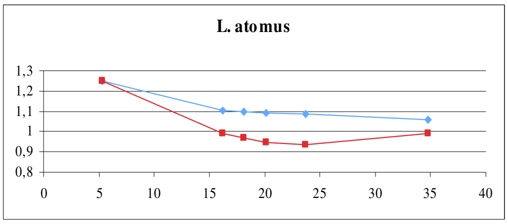

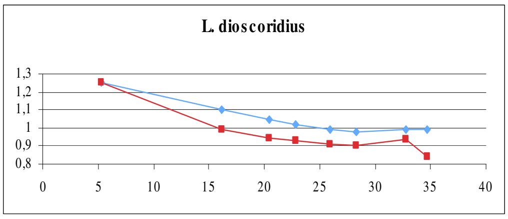

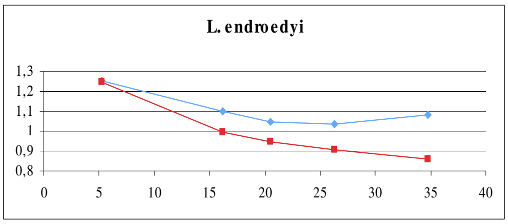

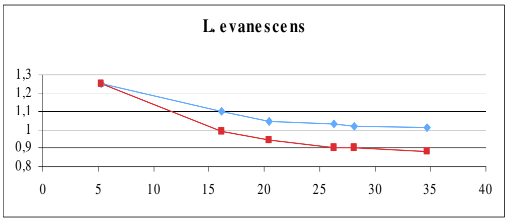

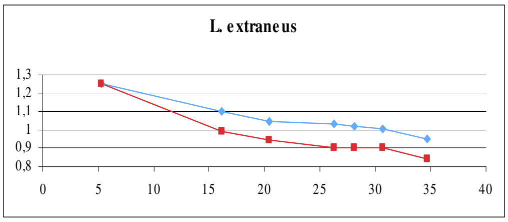

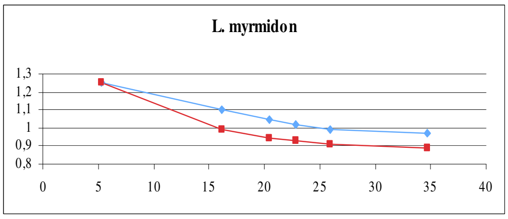

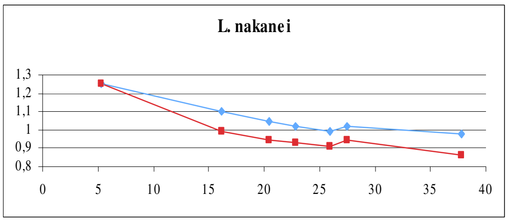

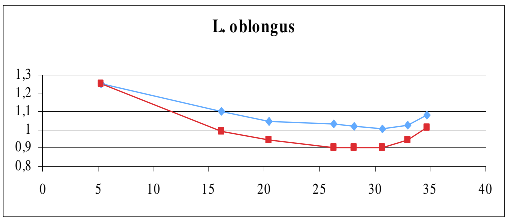

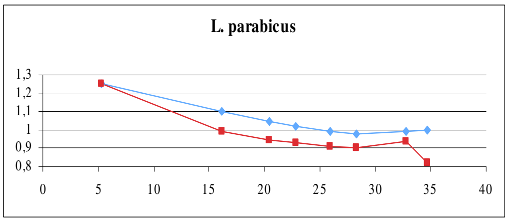

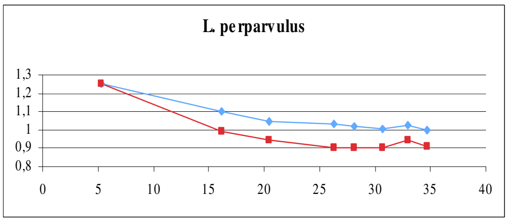

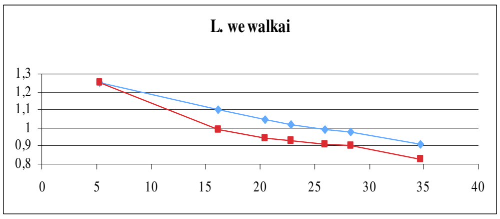

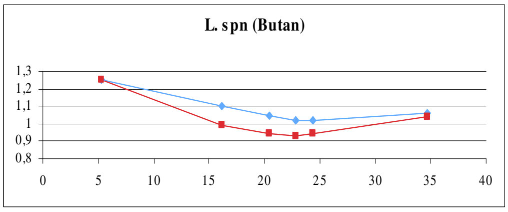

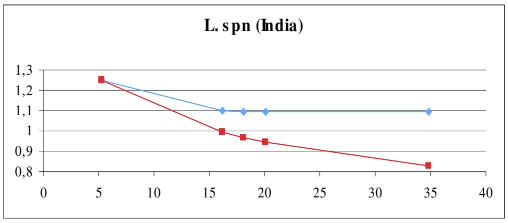

Limnebius sstr

L. parvulus group

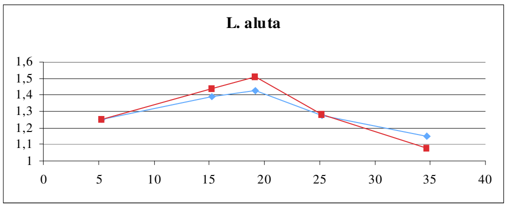

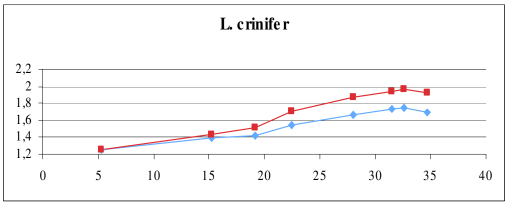

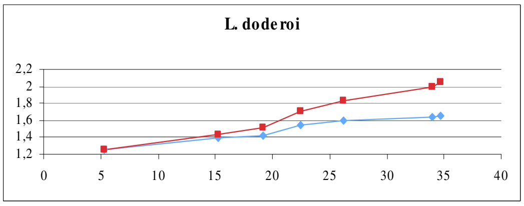

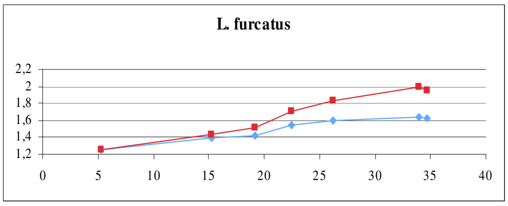

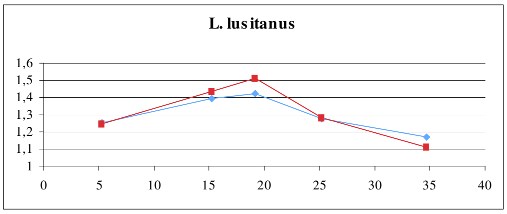

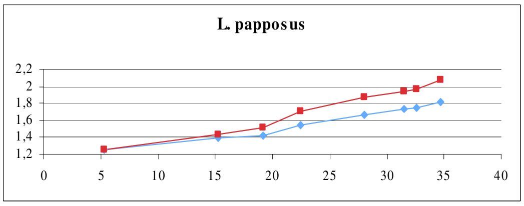

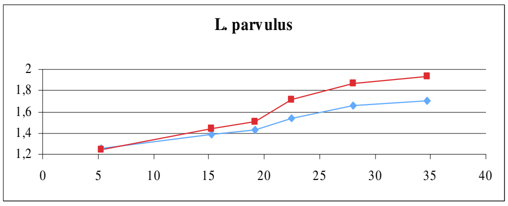

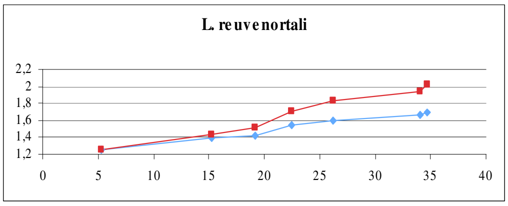

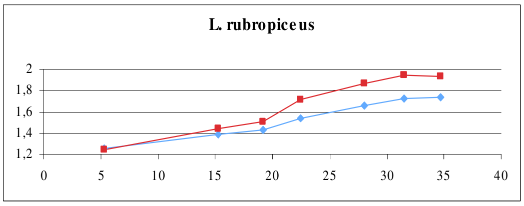

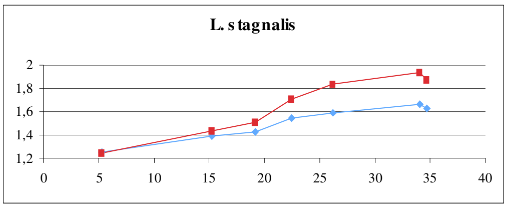

L. piceus group

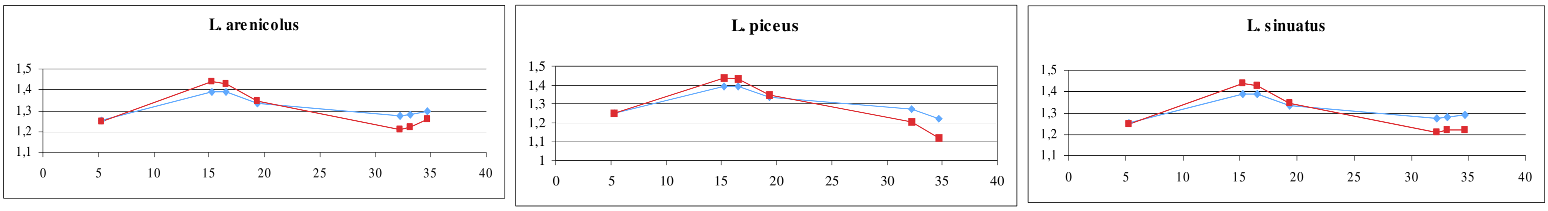

L. gracilipes group

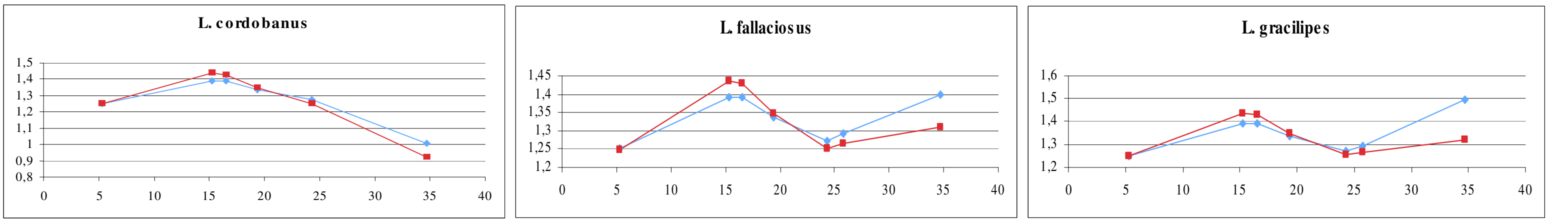

L. nitidus group

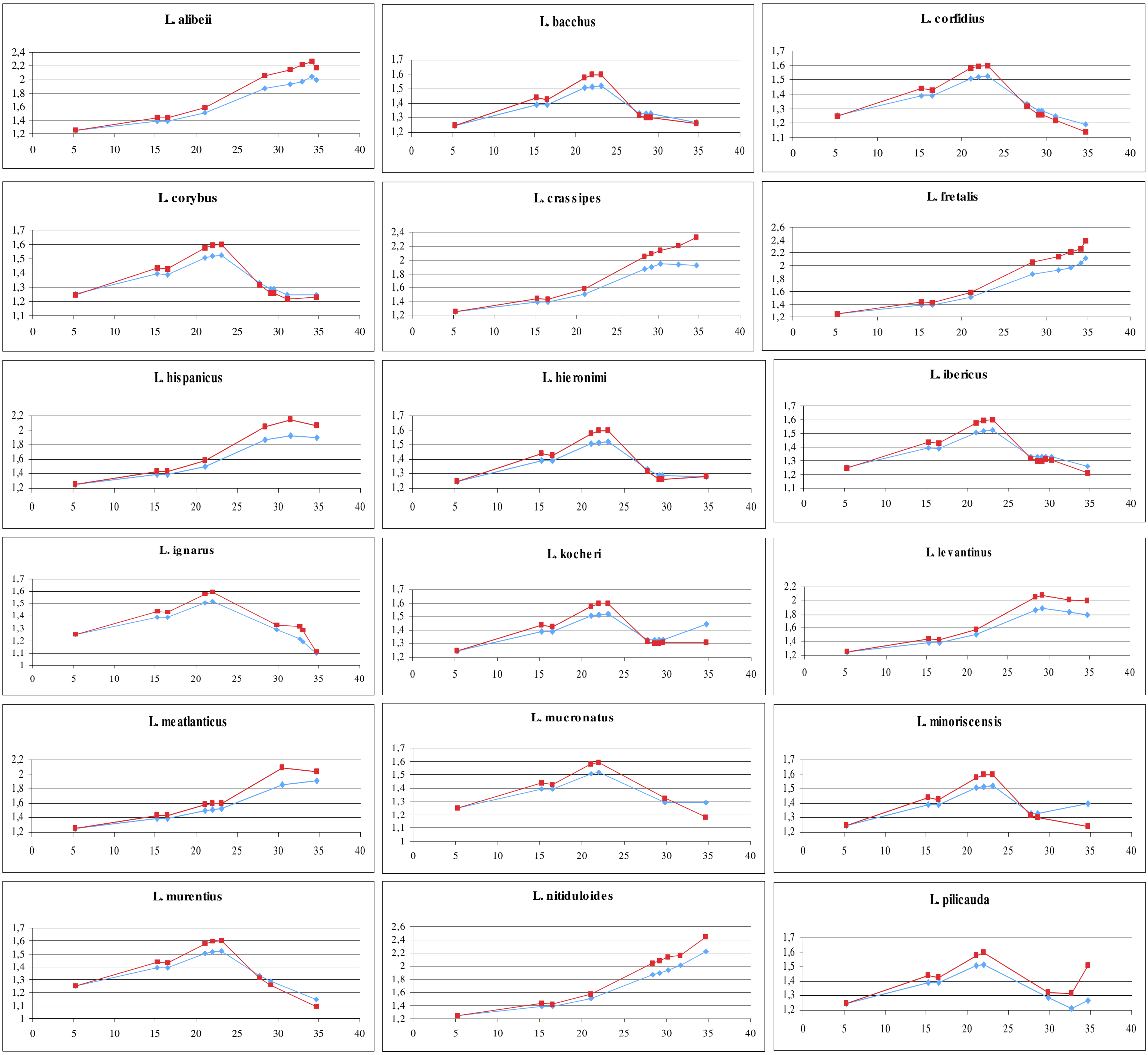

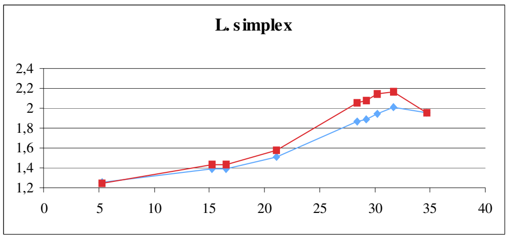
L. simplex
2,4
2,2
2
1,8
1,6
1,4
1,2
0
5
10
15
20
25
30
35
40

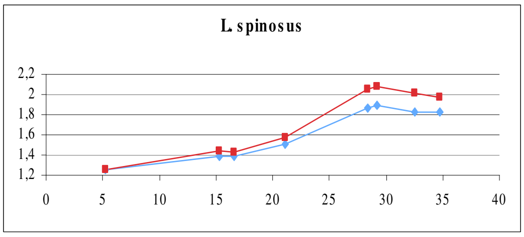
L. spinosus
2,2
2
1,8
1,6
1,4
1,2
0
5
10
15
20
25
30
35
40

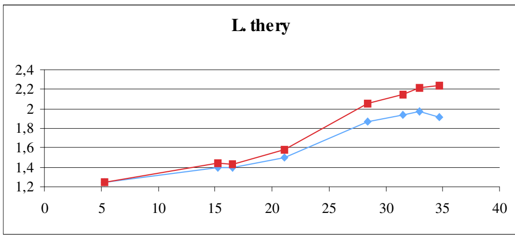
L. thery
2,4
2,2
2
1,8
1,6
1,4
1,2
0
5
10
15
20
25
30
35
40

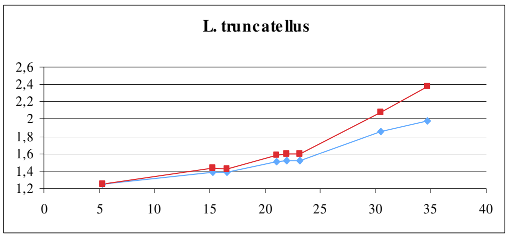
L. truncatellus
2,6
2,4
2,2
2
1,8
1,6
1,4
1,2
0
5
10
15
20
25
30
35
40

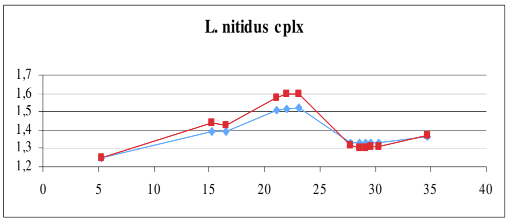
L. nitidus cplx
1,7
1,6
1,5
1,4
1,3
1,2
0
5
10
15
20
25
30
35
40
